# Robotic Assisted Hernia Repair in Four Nordic Countries  - Status and Challenges

**DOI:** 10.3389/jaws.2024.13224

**Published:** 2024-06-27

**Authors:** Frederik Helgstrand, Göran Rietz, Björn Törnqvist, Jan Roland Lambrecht, Robin Gaupset, Tero Rautio, Jaana Vironen

**Affiliations:** ^1^ Zealand University Hospital, Køge, Denmark; ^2^ Stockholm South General Hospital, Karolinska Institutet, Stockholm, Sweden; ^3^ Danderyds Hospital, Karolinska Institutet, Stockholm, Sweden; ^4^ Sykehuset Innlandet Hospital Trust, Hamar, Norway; ^5^ Akershus University Hospital, Nordbyhagen, Norway; ^6^ Oulu University Hospital, Oulu Medical Research Center, Oulu, Finland; ^7^ Helsinki University Hospital, Abdominal Center, Helsinki University, Helsinki, Finland

**Keywords:** hernia, robotic, Nordic, statement, outcome

## Introduction

Hernia is a prevalent medical condition affecting millions of individuals globally. The lifetime risk of undergoing inguinal hernia repair is 27% for men and 3% for women [1]. One in 10 patients will develop an incisional hernia after previous surgery, 1 in 20 will develop an umbilical hernia and up to 50% of all patients with a stoma will develop a parastomal hernia. Consequently, hernia repairs are among the most commonly performed surgical procedures worldwide and even minor improvements can significantly impact healthcare [2].

Traditionally, hernia repair surgery has been conducted using open or laparoscopic techniques.

However, recent registry and single-centre studies have shown that robotic-assistedhernia repair has emerged as a promising alternative, providing benefits such as improved precision, reduced invasiveness, and better outcomes in terms of less pain, fewer wound complications and a shorter length of stay [3–5]. Therefore, the integration of robotic hernia surgery represents a significant opportunity to improve surgical practice and patient experience. Regardless of how promising the tools are, active surveillance is necessary, and the already established hernia registries such as the Danish and Swedish Hernia Registers provide the basis to do so.

In Nordic countries, the adoption of robotic-assisted hernia surgery is increasing, although more slowly compared to other nations. Several challenges persist in the implementation of robotic-assisted hernia repair procedures [6].

To address these challenges a self-organised group of hernia experts from four Nordic countries decided to convene and discuss the future of robotic-assisted hernia surgery from a clinical perspective. The aim of the meetings was to review the current literature and discuss the potential benefits, challenges, and considerations associated with the adoption of robotic-assisted hernia surgery in their countries.

## Methods

This paper was developed through three in-person roundtable discussions, each lasting 2–3 h. Various aspects of robotic hernia surgery were discussed, and the literature was reviewed to validate the statements. The group included dedicated hernia surgeons from Denmark, Finland, Norway, and Sweden, all of whom co-authored this paper. All surgeons have more than 15 years of experience with hernia surgery and 2–7 years of experience with robotic hernia surgery. All surgeons use a Da Vinci system from Intuitive Surgical in their daily practice.

## Present Status of Robotic-Assisted Hernia Surgery in Denmark, Finland, Norway, and Sweden

Currently robotic-assisted hernia surgery constitutes less than 5% of all hernia repairs in the Nordic countries. However, the number of procedures is increasing as additional robotic platforms are installed. More than 120 robotic platforms are currently in place in the Nordic countries. In Denmark nearly all public hospitals now have at least one robotic platform, although none are dedicated solely to hernias. In contrast, other Nordic countries have an uneven distribution of these platforms. Helsinki University Hospital is unique in having a robotic system entirely dedicated to hernia surgery, which has significantly transformed its practice. Early unpublished data from the centre show that since the inception of the robotic programmes 2 years ago the rate of minimally invasive major ventral hernia surgery has increased from 25% to 75%. These data support the idea that all patients undergoing laparoscopic hernia repair and over 50% of those undergoing open hernia repair could benefit from robotic-assisted hernia repair techniques. Therefore, there is a great potential for progress.

## Benefits of Robotic-Assisted Hernia Surgery

### Minimal Invasiveness

Similar to conventional laparoscopic hernia surgery, robotic systems facilitate smaller incisions, resulting in less tissue trauma and a reduced risk of complications such as infection and pain.

### Enhanced Precision

Robotic platforms provide surgeons with greater precision and dexterity compared to traditional laparoscopic techniques. This allows for more accurate dissection and suturing, which is particularly advantageous in complex hernia repairs. Moreover, the free angulation of the instruments and the ability to operate minimally invasively in confined anatomical spaces, enable mesh placement outside the abdominal cavity to avoid adhesions and make it feasible to treat patients with very complex hernias that previously required extensive open surgery.

### Improved Patient Outcomes

Although some studies show no significant differences, data from the Nordic countries indicate that robotic-assisted hernia surgery for ventral hernia reduces postoperative pain, shortens hospital stay, accelerates recovery time and ultimately improves patient satisfaction and quality of life compared to open and laparoscopic approaches; [3, 5, 7–11]. The outcomes of robotic-assisted inguinal hernia repair are excellent and comparable with the traditional laparoscopic methods [12, 13].

### Surgeon Ergonomics

This often-overlooked issue is vital as the retirement age for surgeons rises. Robotic-assisted surgery offers improved visualisation, better ergonomics and instrument control; robotic-assisted surgery also reduces surgeon fatigue and musculoskeletal strain, thereby enhancing overall surgical performance [14, 15].

### Training and Education

Integrating robotic technology into hernia surgery provides optimal training opportunities for the next-generation of surgeons. Robotic platforms elevate surgical simulation, facilitate on-site supervision, and offer an objective evaluation of a surgeon’s development. Options like instant video assessment, remote guidance and re-evaluation support continuous improvement in advanced surgical procedures. Future platforms are likely to integrate artificial intelligence and enhanced imaging, further supporting surgical decision making and facilitating more precise and safer surgeries [16].

## Challenges and Considerations

### Cost

The initial investment and ongoing maintenance costs associated with robotic systems are substantial, raising concerns about cost-effectiveness and resource allocation in healthcare systems. Typically, the focus is on the direct costs to hospitals rather than the total cost of treatment.

In Nordic countries, reimbursement systems incentivise daycare surgeries, shorter hospital stays, and better outcomes. This encourages standardisation, enhanced recovery protocols, minimally invasive treatments, and improved outcomes. The introduction of robotic systems has the potential for even better results, but requires upfront investments at the start of the patient pathway to realise benefits in subsequent stages, including a faster return to work.

These investments are usually covered by the hospitals and the departments executing the surgeries within their semi-fixed budgets. Although there may be a potential overall economic gain over time, the initial costs and the prevailing silo mentality often dominate decision-making.

Nordic healthcare systems are known for their innovation and patient-focused care, financed by taxes and managed by politicians. While there is an awareness of value for money, decisions are also influenced by public opinion and lobbying by patient organisations [17]. High-volume conditions like hernias do not have strong patient organisation representation, although they have a significant impact on health and quality of life. Small improvements in the treatment of hernia patients could greatly benefit healthcare and societal finances, yet investments in new health technologies are typically directed towards treatments that garner political attention. Moreover, the multiple stakeholders, each with their own independent budget, make it difficult to determine the total cost of patient therapy. This complexity inhibits investments that only benefit other stakeholders. Often, pre- and post-surgery sick leave and recovery are not considered, even though they are crucial cost drivers.

In many other surgical procedures such as the treatment of colorectal cancer, robotic platforms have replaced conventional laparoscopic techniques with limited evidence of improved patient outcomes [18]. In hernia surgery, robotic platforms empower surgeons to transform open surgery into minimally invasive surgery and therefore have huge potential for both economic and patient benefit. With many robotic systems already in place in Nordic countries and more on the way, the potential economic savings could easily be capitalised, especially with high utilisation and targeted use.

### Training and Accreditation

Robotic-assisted surgery requires specialised training for surgeons and operating room staff to ensure safe and effective use. Establishing standardised training programmes and accreditation processes is essential for maintaining high-quality care and patient safety, but also to exploit the potential of this tool. The authors predict that robotic platforms will replace traditional laparoscopic and many open surgical procedures for the next-generation of surgeons. Therefore, robotic training programmes for trainees should already be established now, and it is expected that such programmes will be an essential recruitment criterion in the future.

Collaboration between academic institutions, professional societies, and industry partners is crucial for developing comprehensive training programmes for surgeons and surgical teams, ensuring proficiency in robotic-assisted techniques and adhering to best practices.

### Access and Equity

Ensuring equitable access to robotic-assisted hernia surgery across the Nordic countries, including remote and underserved regions, is vital to avoid exacerbating disparities in healthcare delivery and outcomes.

## Discussion

Meetings in which surgeons from different countries discussed and debated the current opportunities and obstacles were an effective method of understanding the present state of robotic-assisted hernia surgery in four countries with similar healthcare systems. The Surgeons all agreed that robotic hernia surgery holds great promise for improving patient care and that Nordic countries have the potential to lead its development. However, limitations and obstacles need addressing. Continued investment in clinical research and initiatives to improve the quality of patient care are necessary to generate robust evidence on the safety, efficacy, and cost benefits of robotic-assisted hernia surgery to guide clinical decision-making and healthcare policy development. The already existing national registries could play a key role in providing essential data.

Furthermore, clear regulatory guidelines and reimbursement policies are needed to govern the use of robotic technology in hernia surgery. Issues such as patient eligibility criteria, procedural volume requirements, and reimbursement rates must be addressed to ensure sustainable adoption and equitable access.

Until recently only one company could provide robotic surgical platforms. Fortunately, several other companies are now entering the market and hopefully more will follow to ensure more competition, more development, lower costs for consumers and more benefits for patients.

Based on the current literature and experience, this group of authors is convinced that robotic platforms will complement conventional laparoscopy and improve surgery in the future.

To ensure the safe and sustainable adoption of robotic-assisted hernia surgery, healthcare institutions and policymakers are encouraged to prioritise investment in robotic surgical infrastructure, including the acquisition of robotic systems, training facilities and ongoing technical support. Collaboration and knowledge exchange between healthcare professionals, industry stakeholders, and patient advocacy groups can foster innovation, promote best practices, and address shared challenges.

## Conclusion

We believe that integrating robotic-assisted hernia surgery will improve the patient experience and the quality of surgical care. However, achieving these benefits requires addressing challenges related to cost, training, access, regulation, and long-term evaluation. Through innovation and collaboration, prioritising patient-centred care and developing existing national hernia registries, Nordic countries can position themselves at the forefront of robotic hernia surgery, benefiting both patients and healthcare systems.
